# SLC2A1 is a Diagnostic Biomarker Involved in Immune Infiltration of Colorectal Cancer and Associated With m6A Modification and ceRNA

**DOI:** 10.3389/fcell.2022.853596

**Published:** 2022-03-24

**Authors:** Xu-Sheng Liu, Jian-Wei Yang, Jing Zeng, Xue-Qin Chen, Yan Gao, Xue-Yan Kui, Xiao-Yu Liu, Yu Zhang, Yao-Hua Zhang, Zhi-Jun Pei

**Affiliations:** ^1^ Department of Nuclear Medicine and Institute of Anesthesiology and Pain, Taihe Hospital, Hubei University of Medicine, Shiyan, China; ^2^ Department of Nuclear Medicine, Xiangyang Central Hospital, Affiliated Hospital of Hubei University of Arts and Science, Xiangyang, China; ^3^ Department of Infection Control, Taihe Hospital, Hubei University of Medicine, Shiyan, China; ^4^ Hubei University of Medicine, Shiyan, China; ^5^ Hubei Clinical Research Center for Precise Diagnosis and Treatment of Liver Cancer, Taihe Hospital, Hubei University of Medicine, Shiyan, China

**Keywords:** SLC2A1, colorectal cancer, immune infiltration, m6A modification, ceRNA

## Abstract

**Background:** Overexpression of solute carrier family 2 member 1 (SLC2A1) promotes glycolysis and proliferation and migration of various tumors. However, there are few comprehensive studies on SLC2A1 in colorectal cancer (CRC).

**Methods:** Oncomine, The Cancer Genome Atlas (TCGA), and Gene Expression Omnibus (GEO) databases were used to analyze the expression of SLC2A1 in pan-cancer and CRC and analyzed the correlation between SLC2A1 expression and clinical characteristics of TCGA CRC samples. The expression level of SLC2A1 in CRC was certified by cell experiments and immunohistochemical staining analysis. The Genome Ontology (GO), Kyoto Encyclopedia of Genes and Genomes (KEGG), and Gene Set Enrichment Analysis (GSEA) analyses of SLC2A1 relative genes were completed by bioinformatics analysis. The correlation between SLC2A1 expression level and CRC immune infiltration cell was analyzed by Tumor IMmune Estimation Resource (TIMER), Gene Expression Profiling Interactive Analysis (GEPIA), and TCGA database. The correlation between SLC2A1 expression level and ferroptosis and m6A modification of CRC was analyzed by utilizing TCGA and GEO cohort. Finally, the possible competing endogenous RNA (ceRNA) networks involved in SLC2A1 in CRC are predicted and constructed through various databases.

**Results:** SLC2A1 is highly expressed not only in CRC but also in many other tumors. ROC curve indicated that SLC2A1 had high predictive accuracy for the outcomes of tumor. The SLC2A1 expression in CRC was closely correlated with tumor stage and progression free interval (PFI). GO, KEGG, and GSEA analysis indicated that SLC2A1 relative genes were involved in multiple biological functions. The analysis of TIMER, GEPIA, and TCGA database indicated that the SLC2A1 mRNA expression was mainly positively associated with neutrophils. By the analysis of the TCGA and GEO cohort, we identified that the expression of SLC2A1 is closely associated to an m6A modification relative gene Insulin Like Growth Factor 2 MRNA Binding Protein 3 (IGF2BP3) and a ferroptosis relative gene Glutathione Peroxidase 4 (GPX4).

**Conclusion:** SLC2A1 can be used as a biomarker of CRC, which is associated to immune infiltration, m6A modification, ferroptosis, and ceRNA regulatory network of CRC.

## Introduction

Recent studies show that colorectal cancer (CRC) is the third most common cancer and the second most deadly in the world (Sung et al., 2021). Despite significant advances in major treatments such as radical resection, radiotherapy, and chemotherapy, the 5-year survival rate for patients with CRC remains low (Chen et al., 2016; Wang et al., 2021). The occurrence, development, metastasis, and recurrence of CRC after treatment involve a variety of important signal transduction pathways in the body, which is a very complex biological process (Shen et al., 2020). Therefore, in-depth investigation of the pathogenesis of CRC can provide better reference strategies for the diagnosis and treatment of tumors.

Solute carrier family 2 member 1 (SLC2A1) encodes a glucose transporter (GLUT) that is the earliest discovery of humans, GLUT1, encoded on 1p34.2 (Kasahara and Hinkle, 1977; Uldry and Thorens, 2004). Multiple projects have indicated that the overexpression of SLC2A1 is closely related to the progression and metastasis of a variety of cancers (Rudlowski et al., 2004; Pereira et al., 2013; Deng et al., 2014; Berlth et al., 2015; Avanzato et al., 2018). SLC2A1 plays an important role in both normal human metabolism and tumor cell glycolysis (Shen et al., 2020). Although it has been found that the overexpression of SLC2A1 can further the glycolysis process and cell proliferation of CRC (Shen et al., 2020; Chen et al., 2021), the biological function of SLC2A1 in CRC has not been extensively studied. Further investigation of other biological functions that SLC2A1 may be involved in CRC will provide a new basis for improving the diagnosis and treatment of CRC.

Tumor immunotherapy (Wang et al., 2014; Ning et al., 2015), N6-methyladenosine (m6A) modification (Bai et al., 2019; Yang et al., 2020), ferroptosis (Ma et al., 2018; Yang et al., 2021), and competing endogenous RNA (ceRNA) network (Zhao et al., 2021) are hot topics of cancer gene therapy, which are broadly utilized in the investigation and therapy of CRC. Nevertheless, there are few research studies on the comprehensive analysis of SLC2A1 in CRC, particularly the relation between SLC2A1 and immune therapy, m6A modification, ferroptosis, and ceRNA regulatory network of CRC.

**FIGURE 1:**
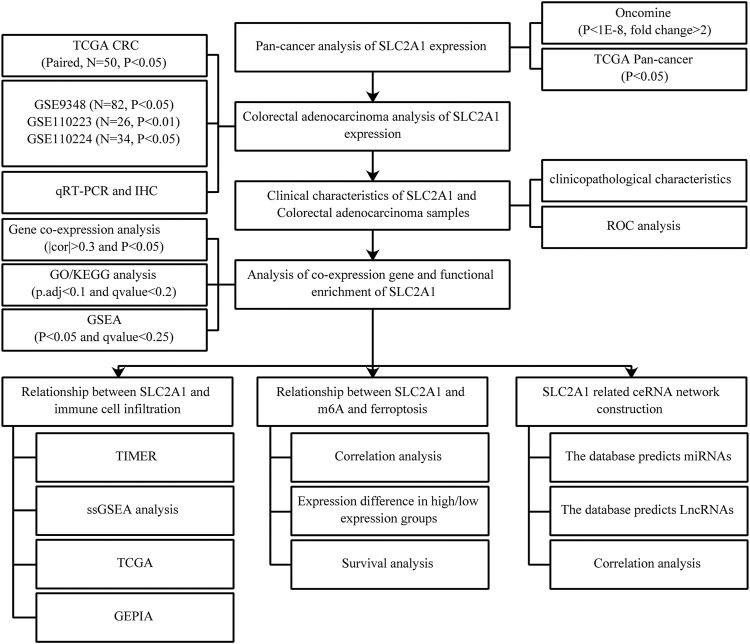
Schematic diagram of the study design.

On this project, we processed The Cancer Genome Atlas (TCGA) CRC and the Gene Expression Omnibus (GEO) CRC cohort *via* an online network. Bioinformatics analysis was carried utilizing R language, analysis tools, and online website to research the difference of SLC2A1 expression in pan-cancer, and *in vitro* experiments and immunohistochemical (IHC) staining were performed to confirm the difference of SLC2A1 mRNA and protein expression between CRC and normal samples. At present, the SLC2A1 co-expression gene networks in CRC were analyzed and the possible biological function and signal regulation pathway involved in these relative genes were investigated. Eventually, the relation between SLC2A1 and CRC tumor ferroptosis, immunofiltration, m6A modification, and ceRNA network was investigated, which provides a basis for the development of new therapeutic strategies. The schematic diagram of the research design is shown in Figure 1.

## Materials and Methods

### Expression of SLC2A1 in CRC

Oncomine online database (www.oncomine.org) (Rhodes et al., 2004; Rhodes et al., 2007) and TCGA cohort (https://portal.gdc.cancer.gov) (Tomczak et al., 2015) were used to analyze the expression differences of SLC2A1 in varied cancers. Student’s t-test was used to compare the expression difference of SLC2A1 in tumor and control samples in Oncomine database, and data with fold change >2 and P < 1E-8 were selected for display. In the TCGA cohort, the CRC cohort used included the colon adenocarcinoma (COAD) and rectum adenocarcinoma (READ) datasets. We also analyzed CRC cohort from the TCGA and GEO (www.ncbi.nlm.nih.gov/geo; GSE9348, GSE110223, and GSE110224) (Barrett et al., 2012) databases to investigate differences in SLC2A1 expression level between tumor and control samples. By analyzing the clinical datasets of TCGA CRC cohort, the relation between SLC2A1 expression and clinicopathological characteristics of patients with CRC was investigated, and the diagnostic value of SLC2A1 for CRC was evaluated by ROC curve. Eventually, we certified the difference in SLC2A1 expression between CRC and normal samples by used qRT-PCR and IHC staining. Refer to our previous work (Liu et al., 2021a) for specific steps, including specific methods of qRT-PCR and IHC staining, as detailed in the Supplementary Material.

### SLC2A1 Gene Co-Expression Network and Enrichment Analysis in CRC

The TCGA CRC cohort was analyzed utilizing the R statistical computing language (version 3.6.3) to investigate co-expressed genes related with SLC2A1 expression. The Pearson’s correlation coefficient was used for statistical analysis, and correlations were considered as significant when |cor| > 0.3 and *p* < 0.05. Ggplot2 software package of R language (https://ggplot2.tidyverse.org) was utilized to draw volcano and heat map for demonstration. Top 200 genes that were positively correlated with SLC2A1 expression were selected as hub genes. The Genome Ontology (GO) term and Kyoto Encyclopedia of Genes and Genomes (KEGG, http://www.genome.jp/kegg) pathway investigation of hub genes was carried by using the clusterProfiler (Yu et al., 2012) software package of R language, and the data were analyzed by using the ggplot2 software package.

### Gene Set Enrichment Analysis

To study the potential function of SLC2A1 in CRC, we divided the samples in the CRC cohort into high and low groups based on the SLC2A1 median expression in CRC and carried Gene Set Enrichment Analysis (GSEA) (www.gsea-msigdb.org/gsea/index.jsp) (Subramanian et al., 2005) to research whether genes in the two groups were enriched with meaningful biological functions. The annotated gene set c2.cp.v7.2.symbols.gmt (Curated) was selected as the reference gene set. The FDR (q-value) < 0.25 and *p* < 0.05 were considered statistically significant.

### Associations Between SLC2A1 and Tumor Immune Infiltrating Cells

To investigate the possible regulatory mechanism of SLC2A1 in the regulation of CRC immune cells, we utilized the TIMER database (https://cistrome.shinyapps.io/timer) to evaluate the relationship between SLC2A1 expression and immunofiltration cells in TCGA CRC samples. Immunofiltration cells include B cell, neutrophil, CD4+ T cell, CD8+ T cell, macrophage, and dendritic cell (DC). We utilized the somatic copy number alteration module of the TIMER tool to correlate genetic copy number variation (CNV) of SLC2A1 with the relative proportion of immune infiltrating cells. The GSVA (Hänzelmann et al., 2013) software package of R language was utilized to analyze the relative abundance of 24 immune cells in CRC samples with high and low SLC2A1 expression, and the specific algorithm was ssGSEA. Furthermore, we analyzed the relation between SLC2A1 and immunofiltration cell marker genes in CRC samples utilizing Tumor IMmune Estimation Resource (TIMER), Gene Expression Profiling Interactive Analysis (GEPIA), and TCGA databases. Immunofiltration marker genes refer to the previous study, in which immune markers of different immune cells are described (Liu et al., 2021b).

### Associations of SLC2A1 Expression Level With m6A Modification in CRC

The association between the expression level of SLC2A1 and the m6A relative genes, including YTHDF1, WTAP, RBM15, FTO, ALKBH5, ZC3H13, YTHDF3, HNRNPC, YTHDC2, METTL14, METTL3, IGF2BP3, IGF2BP2, RBMX, RBM15B, IGF2BP1, YTHDC1, HNRNPA2B1, VIRMA, and YTHDF2 (Li et al., 2019), in GSE110224 and TCGA CRC cohort was analyzed utilizing the R statistical computing language. In the TCGA cohort, the CRC cohort used included the COAD and READ datasets. The ratio of m6A relative genes in CRC samples with high and low SLC2A1 expression was analyzed by R statistical computing language. The Kaplan–Meier curve indicated the relation between the expression level of relative genes and the prognosis of patients with CRC. Ggplot2 software package was utilized for visual analysis of the cohort.

### Associations of SLC2A1 Expression Level With Ferroptosis in CRC

The R statistical computing language was utilized to analyze the association between the expression level of SLC2A1 and ferroptosis relative genes in the GSE110224 and TCGA CRC cohort, including CDKN1A, HSPA5, EMC2, SLC7A11, NFE2L2, MT1G, HSPB1, GPX4, FANCD2, CISD1, FDFT1, SLC1A5, SAT1, TFRC, RPL8, NCOA4, LPCAT3, GLS2, DPP4, CS, CARS, ATP5MC3, ALOX15, ACSL4, and AIFM2 (Doll et al., 2019; Liu et al., 2020). In the TCGA cohort, the CRC cohort used included the COAD and READ datasets. R statistical computing language was utilized to analyze the ratio of ferroptosis relative genes in CRC samples with high and low SLC2A1 expression. The Kaplan–Meier curve indicated the relation between the expression level of relative genes and the prognosis of patients with CRC. Ggplot2 software package was used for visualizing analysis of the cohort.

### Prediction and Construction of SLC2A1 ceRNA Regulatory Network in CRC

The mirDIP (www.ophid.utoronto.ca/mirDIP/) (Tokar et al., 2018), micorT CDS (www.microrna.gr/microT-CDS) (Paraskevopoulou et al., 2013), and miRNet (https://www.mirnet.ca/miRNet/home.xhtml) (Chang et al., 2020) tools were used to predict the miRNAs of target SLC2A1, and the correlation between SLC2A1 and these miRNAs was analyzed in the TCGA CRC cohort to screen negatively correlated miRNAs as target miRNAs. miRNet and starBase (www.starbase.sysu.edu.cn/starbase2/index.php) (Li et al., 2014) tools were used to predict the long non-coding RNA (lncRNAs) of target microRNA (miRNAs), and the correlation between the target miRNAs and these lncRNAs was analyzed in the TCGA CRC dataset, and the negatively correlated lncRNAs were selected as target lncRNAs. To further narrow the prediction range, the expression level of target lncRNA in CRC and its correlation with SLC2A1 were further analyzed according to the ceRNA theory. The igraph software package of R language (http://igraph.org) is used to analyze the data visually.

### Statistical Methods

All statistical analysis was conducted through Xiantao platform (www.xiantao.love). Xiantao platform is a database integrating TCGA tumor microarray data, which is mainly used for gene expression analysis, correlation analysis, enrichment analysis, interactive network analysis, clinical significance analysis, and related plotting.

## Results

### Pan-Cancer Analysis of SLC2A1 mRNA Expression Level in Different Cohort

**FIGURE 2:**
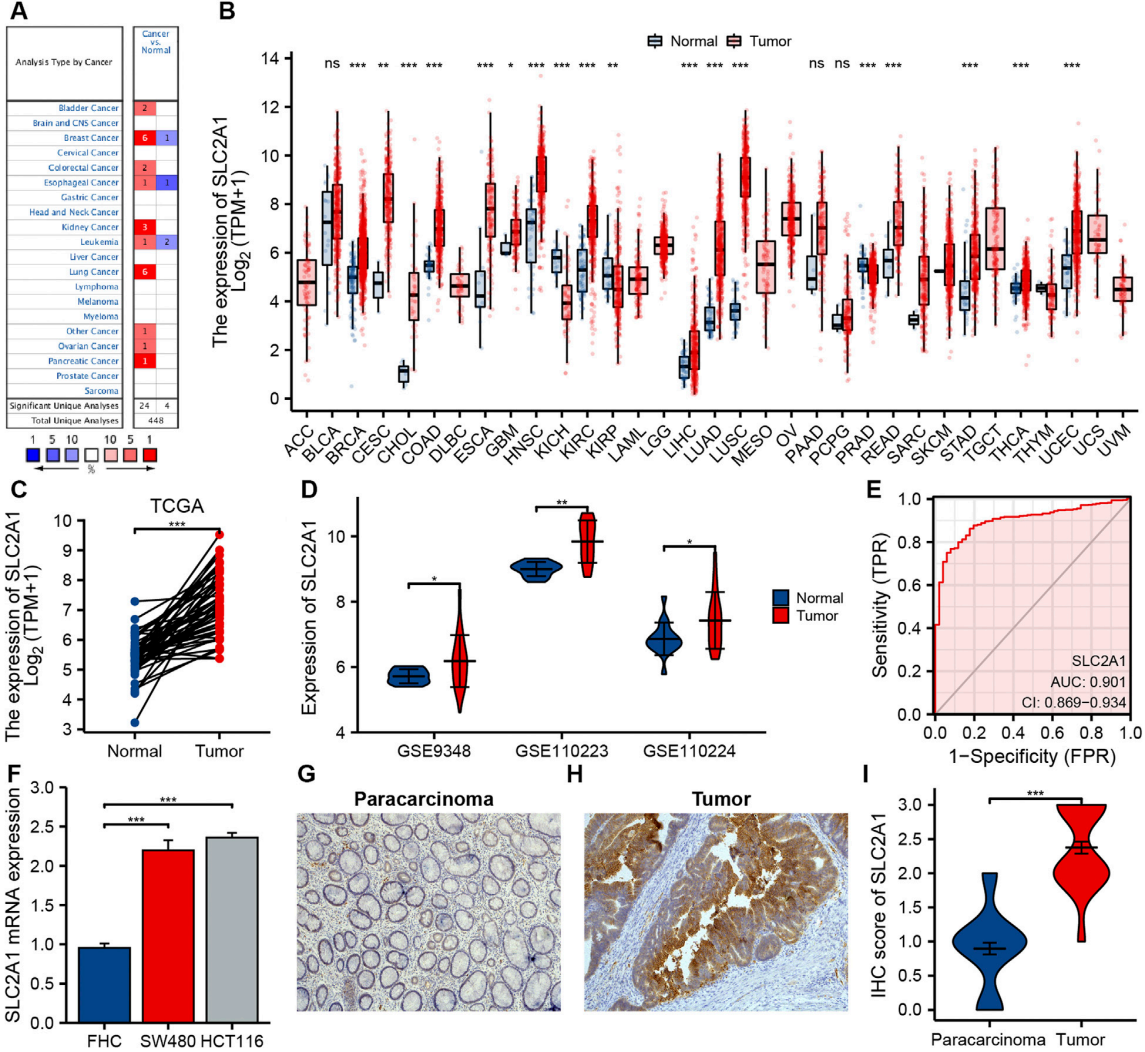
The expression of SLC2A1 in colorectal cancer (CRC) and pan-cancer. **(A)** Oncomine database summarizes the expression of SLC2A1 in pan-cancer. **(B)** TCGA cohort summarizes the expression of SLC2A1 in pan-cancer. **(C)** Matched tumor/normal human CRC specimens showed increased expression of SLC2A1 in tumor specimens in TCGA cohort. **(D)** The GSE9348, GSE110223, and GSE110224 cohort showed an elevated expression of SLC2A1 in tumor specimens. **(E)** ROC curve analysis of SLC2A1 diagnosis. **(F)** Difference of expression of SLC2A1 in CRC cell lines and human normal colorectal mucosa cell lines. Immunohistochemistry assay was used to analyze the protein expression of SLC2A1 in paracarcinoma samples **(G)** and CRC samples **(H)**. **(I)** The mean SLC2A1 IHC score in CRC samples was remarkably higher than that of matched paracarcinoma samples. *, *p* < 0.05; **, *p* < 0.01; ***, *p* < 0.001; ****, *p* < 0.0001; ns, no significant.

The differences in SLC2A1 mRNA expression level between CRC and control group were analyzed by using Oncomine database and TCGA cohort. Oncomine database analysis indicated that SLC2A1 expression was higher in bladder cancer (Sanchez-Carbayo et al., 2006), breast cancer (Zhao et al., 2004; Curtis et al., 2012), CRC (Ki et al., 2007; Skrzypczak et al., 2010), esophageal cancer (Su et al., 2011), kidney cancer (Jones et al., 2005; Beroukhim et al., 2009), leukemia (Andersson et al., 2007), lung cancer (Su et al., 2007; Landi et al., 2008; Hou et al., 2010; Okayama et al., 2012; Selamat et al., 2012), ovarian cancer (Yoshihara et al., 2009), and pancreatic cancer (Pei et al., 2009) than in normal tissues. Some studies also found that the expression of SLC2A1 in breast cancer (Finak et al., 2008), esophageal cancer (Kim et al., 2010), and leukemia (Haferlach et al., 2010) was lower than that in normal samples (Figure 2A). Table 1 summarizes the details of SLC2A1 expression levels in pan-cancers.

**TABLE 1:** SLC2A1 expression in cancerous versus normal tissue in ONCOMINE.

Cancer Site	Cancer Type	*p* Value	t-Test	Fold Change	Reference (PMID)
Bladder	Infiltrating Bladder Urothelial Carcinoma	1.85E-12	7.682	2.514	16432078
	Superficial Bladder Cancer	1.20E-14	10.564	3.977	16432078
Breast	Invasive Ductal Breast Carcinoma	1.03E-11	9.276	2.800	15034139
	Intraductal Cribriform Breast Adenocarcinoma	2.50E-9	11.263	2.172	TCGA Breast
	Invasive Ductal Breast Carcinoma	4.19E-27	13.974	2.557	TCGA Breast
	Invasive Breast Carcinoma	8.98E-15	8.629	2.251	TCGA Breast
	Medullary Breast Carcinoma	4.82E-10	8.059	2.728	22522925
	Mucinous Breast Carcinoma	6.44E-13	8.635	2.100	22522925
	Invasive Breast Carcinoma	4.56E-17	−11.709	−3.780	18438415
Colorectal	Colon Adenocarcinoma	1.22E-13	8.619	2.107	17640062
	Colorectal Carcinoma	1.64E-9	7.030	2.372	20957034
Esophageal	Esophageal Squamous Cell Carcinoma	6.18E-13	8.162	2.134	21385931
	Barrett’s Esophagus	1.57E-10	−9.953	−3.261	21152079
Kidney	Non-Hereditary Clear Cell Renal Cell Carcinoma	9.35E-13	11.104	5.519	19470766
	Hereditary Clear Cell Renal Cell Carcinoma	7.36E-13	13.447	5.810	19470766
	Clear Cell Renal Cell Carcinoma	1.63E-12	11.171	2.912	16115910
Leukemia	B-Cell Acute Lymphoblastic Leukemia	1.54E-11	11.384	3.218	17410184
	Pro-B Acute Lymphoblastic Leukemia	4.74E-21	−11.337	−2.608	20406941
	T-Cell Acute Lymphoblastic Leukemia	4.15E-23	−12.601	−2.714	20406941
Lung	Lung Adenocarcinoma	1.12E-10	8.781	3.160	17540040
	Lung Adenocarcinoma	2.39E-23	14.833	5.153	22613842
	Squamous Cell Lung Carcinoma	1.92E-23	26.718	22.600	20421987
	Lung Adenocarcinoma	2.68E-14	10.393	4.801	20421987
	Lung Adenocarcinoma	5.86E-21	13.641	2.843	22080568
	Lung Adenocarcinoma	2.15E-16	10.850	2.086	18297132
Ovarian	Ovarian Serous Adenocarcinoma	7.49E-10	11.221	7.952	19486012
Pancreatic	Pancreatic Carcinoma	6.24E-19	14.773	8.453	19732725

We further analyzed SLC2A1 mRNA expression level in different human cancers utilizing TCGA cohort. Figure 2B indicates the differences between SLC2A1 in varied tumor samples and control samples. SLC2A1 expression level in breast invasive carcinoma, cervical squamous cell carcinoma and endocervical adenocarcinoma, cholangiocarcinoma, COAD, esophageal carcinoma, glioblastoma multiforme, head and neck squamous cell carcinoma, kidney renal clear cell carcinoma, kidney renal papillary cell carcinoma, liver hepatocellular carcinoma, lung adenocarcinoma, lung squamous cell carcinoma, READ, stomach adenocarcinoma, thyroid carcinoma, and uterine corpus endometrial carcinoma was remarkably higher than that in control tissues and significantly decreased in kidney chromophobe and prostate adenocarcinoma.

### Expression Levels of SLC2A1 in Patients with CRC

By analyzing CRC datasets from TCGA and GEO, we further determined the difference in SLC2A1 expression between CRC and normal samples. The results of TCGA and GEO cohort analysis demonstrated that the expression level of SLC2A1 remarkably increased in CRC compared to the control group (Figures 2C,D). To further confirm the precision of the analysis results, we performed the qRT-PCR and IHC staining experiments for further verification. As shown in Figure 2F, qRT-PCR results indicated that SLC2A1 mRNA expression remarkably increased in human CRC cell lines SW480 (2.197 ± 0.127 vs. 0.954 ± 0.058) and HCT116 (2.360 ± 0.061 vs. 0.954 ± 0.058) compared with normal human colorectal mucosa cells. The IHC staining results indicated that SLC2A1 was primarily expression in the CRC cell membrane. SLC2A1 IHC scores in tumor tissues were remarkably higher than those in paracancerous tissues (2.375 ± 0.606 vs. 0.896 ± 0.592, Figures 2G–I). These results point out that overexpression of SLC2A1 may conduce to the development of CRC. To estimate the diagnostic ability of SLC2A1 in CRC, we carried the ROC curve analysis. The ROC curve indicated that SLC2A1 had high accuracy in predicting the outcomes of normal and tumor (Figure 2E), and the area under the ROC curve was 0.901 (95% CI: 0.869–0.934).

**FIGURE 3:**
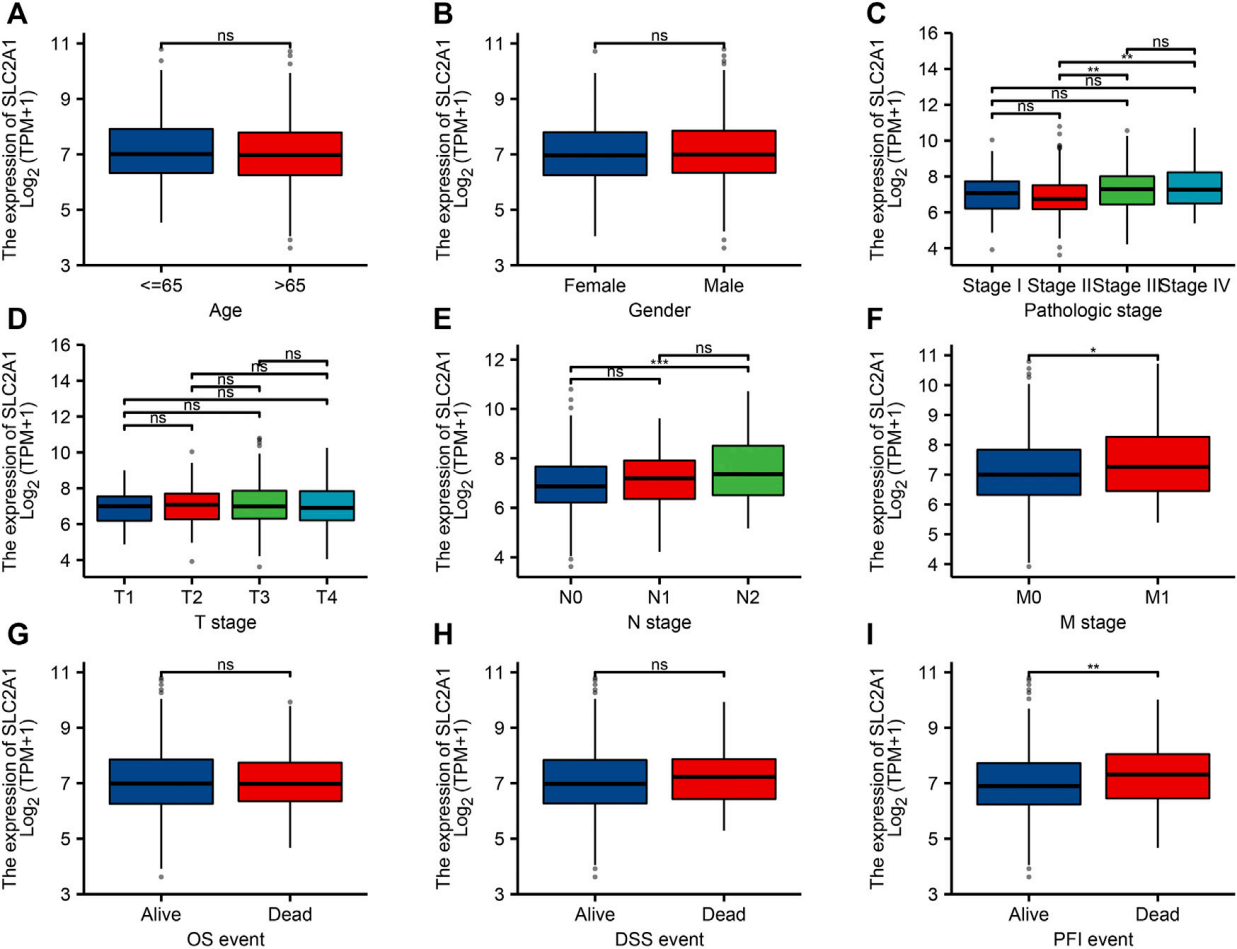
Relationship between SLC2A1 mRNA expression and clinicopathological parameters in patients with colorectal cancer (CRC). The SLC2A1 mRNA expression level was expressed by utilizing ggplot2 software package of R language for the patient characteristics of **(A)** age, **(B)** gender, **(C)** pathologic stage, **(D)** T stage, **(E)** N stage, **(F)** M stage, **(G)** OS event, **(H)** DSS event, and **(I)** PFI event. *, *p* < 0.05; **, *p* < 0.01; ***, *p* < 0.001; ****, *p* < 0.0001; ns, no significant.

To confirm the significance of SLC2A1 in clinical environment, we analyzed clinical data from TCGA CRC cohort. As shown in Figure 3, the results showed that the SLC2A1 expression in Stage II group was lower than that in Stage III and Stage IV groups. SLC2A1 expression in N0 group was lower than that in N2 group. SLC2A1 expression in M0 group was lower than that in M1 group. During progression-free survival events, SLC2A1 expression levels in patients who died were significantly higher than those in the surviving group.

### SLC2A1 Gene Co-Expression Network and Enrichment Analysis in CRC

**FIGURE 4:**
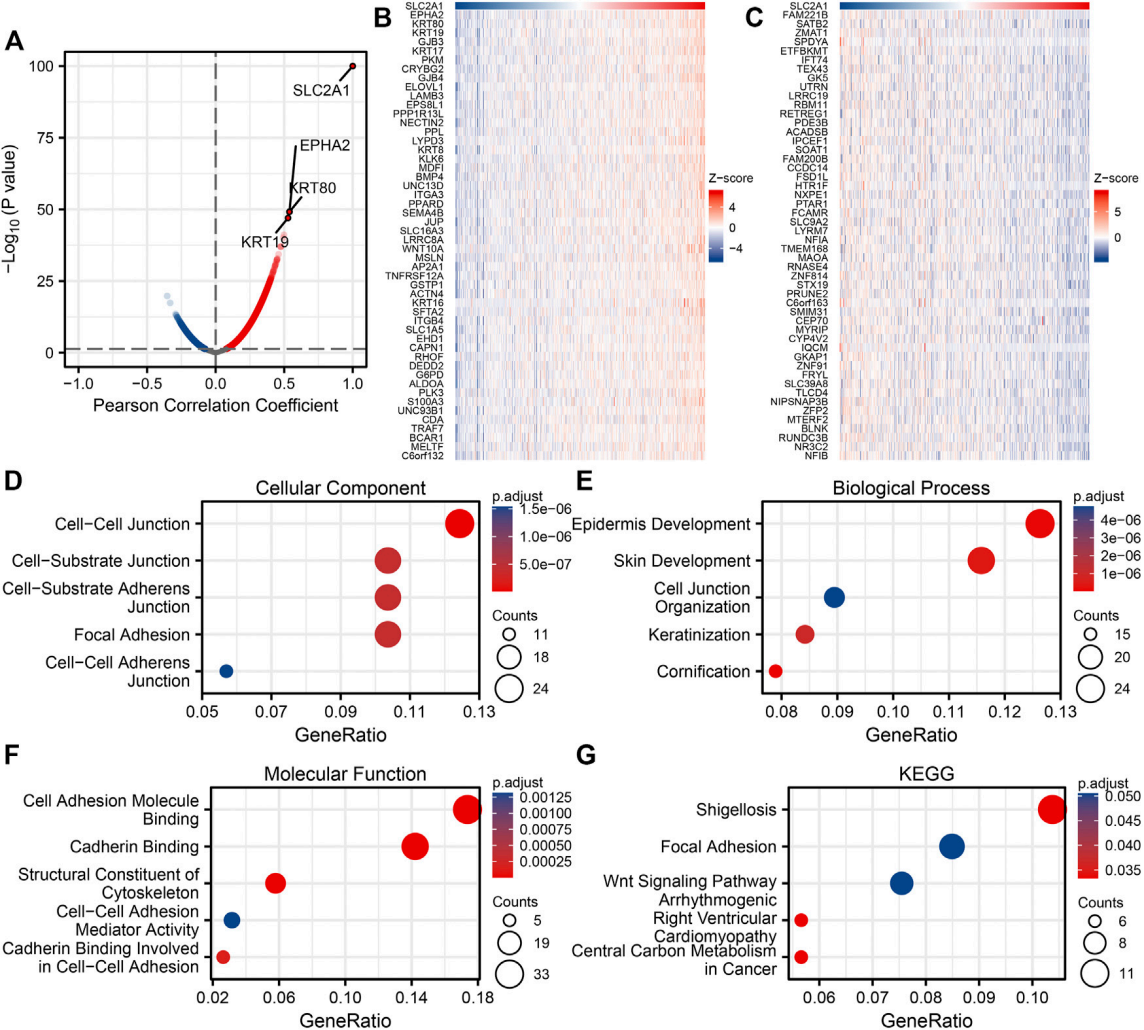
Enrichment analysis of SLC2A1 gene co-expression network in colorectal cancer (CRC). **(A)** Volcano map indicated co-expression genes correlated with expression level of SLC2A1 in TCGA CRC cohort. **(B, C)** Heat maps indicated the top 50 co-expression genes positively and negatively associated with expression level of SLC2A1 in the TCGA CRC cohort. **(D–F)** Enrichment analysis of gene ontology (GO) terms for SLC2A1 co-expression genes. **(G)** Enrichment analysis of Kyoto Encyclopedia of Genes and Genomes (KEGG) terms for terms for SLC2A1 co-expression genes.

We analyzed co-expressed genes related to SLC2A1 expression in the TCGA CRC dataset using the R statistical computing language. Only the protein-coding genes were kept. The main results presented in Figure 4A genes were positively related with SLC2A1 expression, whereas 3,911 genes were negatively related with SLC2A1 expression (*p* < 0.05). Under the conditions of |cor| > 0.3 and *p* < 0.05, a total of 515 genes were obtained, including 513 positively related genes and two negatively related genes. When the threshold values were cor > 0.5 and *p* < 0.05, the three genes had the strong association: EPHA2 (cor = 0.538, *p* = 6.36E-50), KRT80 (cor = 0.538, *p* = 7.33E-50), and KRT19 (cor = 0.528, *p* = 9.93E-48), respectively. The results, as shown in Figures 4B,C, indicated that the top 50 genes are positively and negatively associated with SLC2A1 expression, respectively. The detailed description of co-expressed genes is indicated in Supplementary Table S1.

The GO term and KEGG pathway investigation of the first 200 co-expressed genes mainly associated with SLC2A1 expression were carried through using the clusterProfiler software package of R language. SLC2A1 co-expressed genes involved 242 biological processes, 80 cell components, 31 molecular functions, and 18 KEGG under the condition of p.adj <0.1 and q-value <0.2. The bubble graph shows the top five messages of biological processes, cell components, molecular functions, and KEGG, respectively. The GO term annotation indicated that these genes were primarily participated in epidermis development, cell–cell junction, and cadherin binding (Figures 4D–F). The KEGG pathway analysis indicated that these genes were chiefly participated in Shigellosis and Wnt signaling pathway (Figure 4G). Supplementary Table S2 summarizes the GO term and KEGG pathway details of SLC2A1 co-expression enrichment analysis.

### Gene Set Enrichment Analysis

**FIGURE 5:**
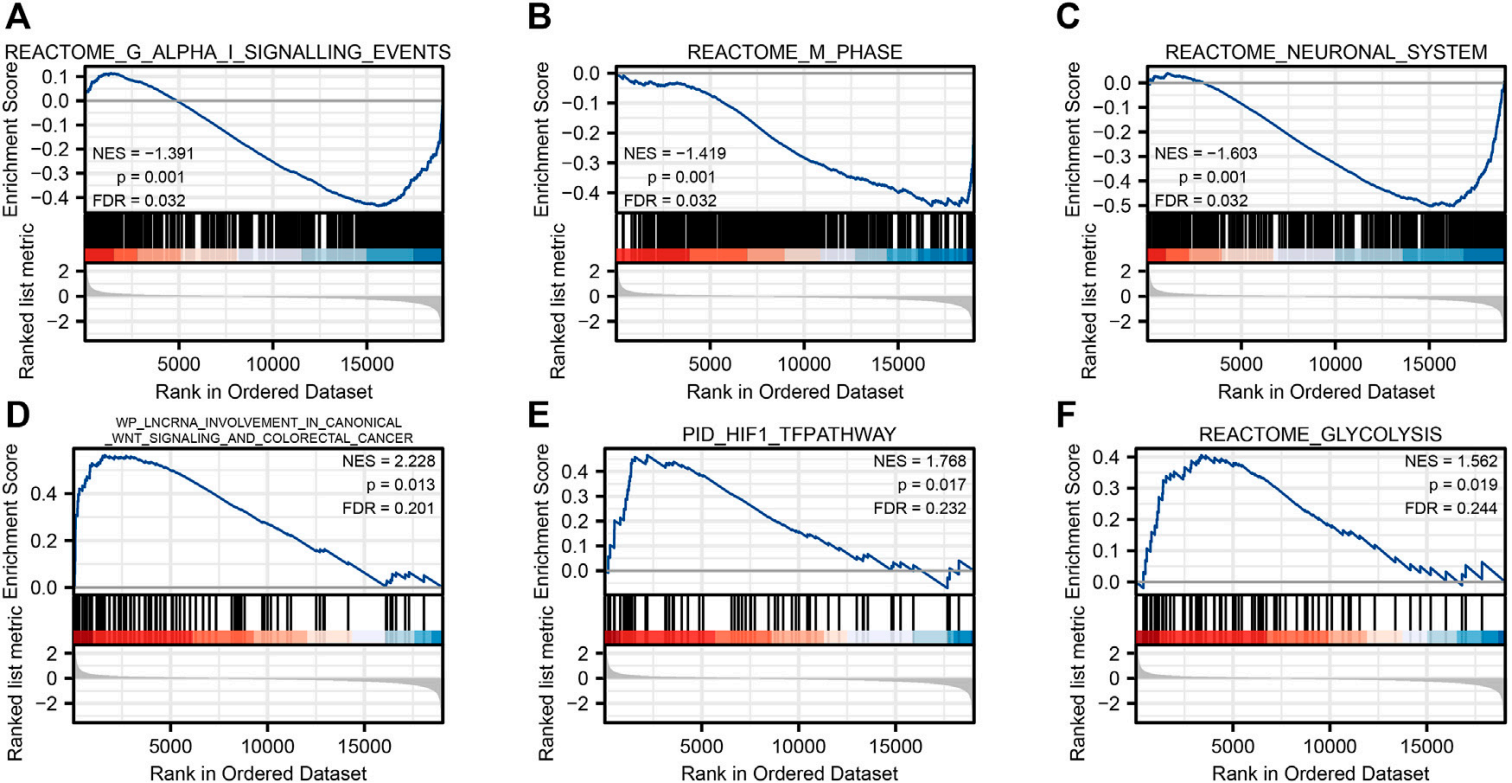
Gene set enrichment analysis. Reactome G alpha I signaling events **(A)**, reactome m phase **(B**), reactome neuronal system **(C)**, WP lncrna involvement in canonical Wnt signaling and colorectal cancer **(D)**, PID HIF1 TFPATHWAY **(E)**, and reactome glycolysis **(F)**.

To investigate the possible mechanism of SLC2A1 in CRC, the GSEA analysis was carried on the differential genes. A total of 194 gene sets were found, among which the top three enrichment pathways with the strongest correlation were REACTOME G ALPHA I SIGNALING EVENTS (FDR = 0.031, *p* = 0.001), REACTOME M PHASE (FDR = 0.031, *p* = 0.001), and REACTOME NEURONAL SYSTEM (FDR = 0.031, *p* = 0.001). At the same time, we also found that the different genes were involved in WP LNCRNA INVOLVEMENT IN CANONICAL WNT SIGNALING AND COLORECTAL CANCER (FDR = 0.200, *p* = 0.013), PID HIF1 TFPATHWAY (FDR = 0.232, *p* = 0.017), and REACTOME GLYCOLYSIS (FDR = 0.244, *p* = 0.019), respectively (Figure 5). Detailed enrichment analysis information is indicated in Supplementary Table S3.

### Associations Between SLC2A1 and Tumor Immune Infiltrating Cells

**FIGURE 6:**
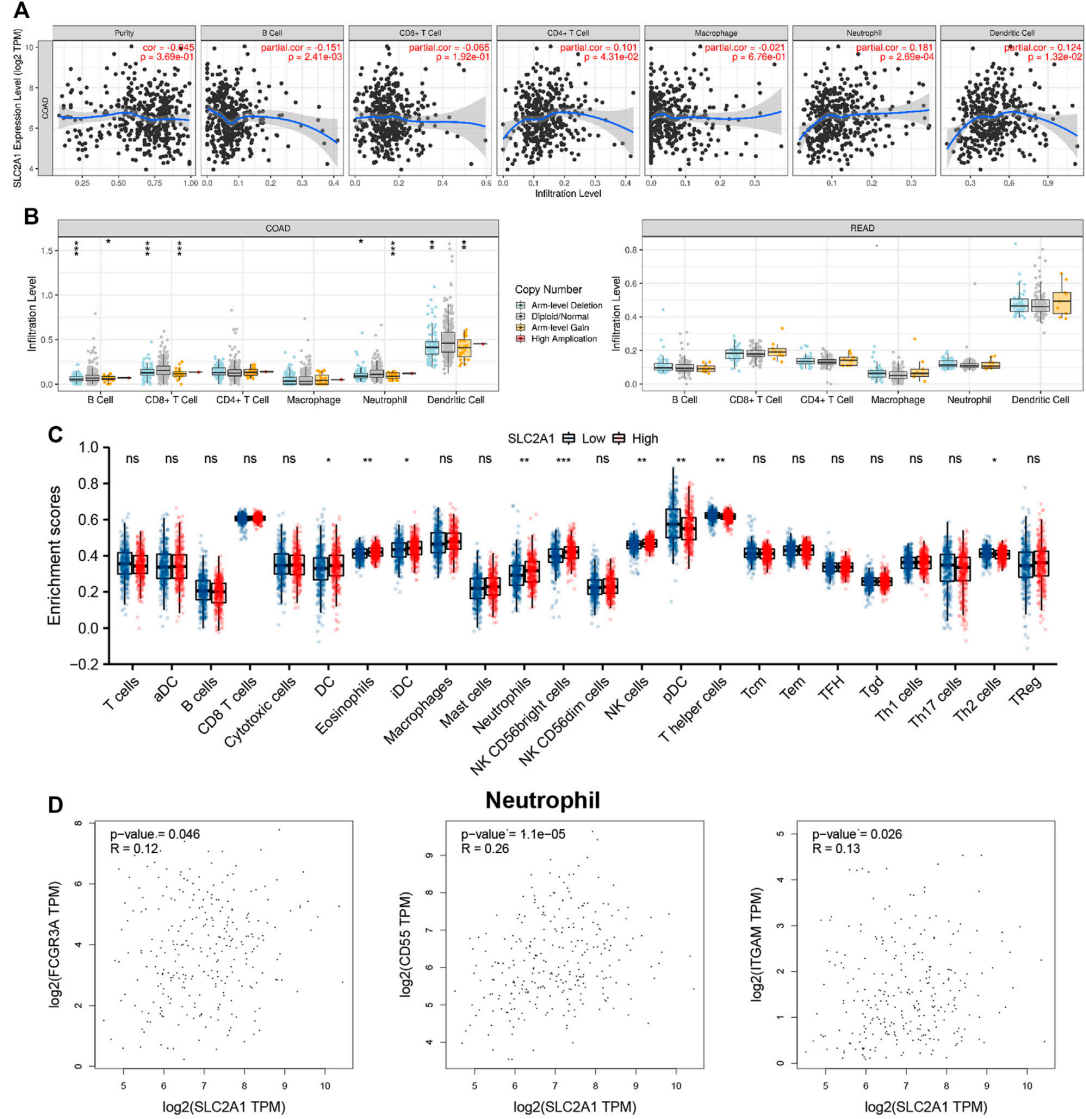
Associations between SLC2A1 and Tumor Immune Infiltrating Cells. **(A)** Association between the expression level of SLC2A1 and immunofiltration cells in colorectal cancer (CRC). **(B)** SLC2A1 CNV affects the infiltrating levels of B cell, CD4+ T cell, macrophages, neutrophils, and dendritic cell in CRC. **(C)** Changes of 24 immune cell subtypes between high and low SLC2A1 expression groups in CRC tumor samples. **(D)** SLC2A1 expression correlated with neutrophils in CRC. *, *p* < 0.05; **, *p* < 0.01; ***, *p* < 0.001; ****, *p* < 0.0001; ns, no significant.

The association between SLC2A1 expression level and CRC samples immunofiltration cells was analyzed by TIMER database. The results indicated that the expression level of SLC2A1 was positively associated with the infiltrating level of CD4+ T cell (r = 0.101, *p* = 4.31E-2), neutrophil (r = 0.181, *p* = 2.69E-4), and DC (r = 0.124, *p* = 1.32E-2) and negatively associated with the infiltrating level of B cell (r = –0.151, *p* = 2.41E-3) (Figure 6A). In addition, SLC2A1 CNV was found to be significantly correlated with the infiltration levels of B cell, neutrophil, CD8+ T cell, and DC in COAD cohort (Figure 6B).

The infiltration abundance of immune cells between the high and low SLC2A1 expression group was analyzed by ssGSEA algorithm, and the results showed that there were differences in the expressions of various immune cells between the two groups (Figure 6C), including DC (*p* = 0.012), eosinophils (*p* = 0.001), immature DC (iDC; *p* = 0.032), neutrophils (*p* = 0.001), natural killer (NK) CD56 bright cells (*p* < 0.001), NK cells (*p* = 0.002), plasmacytoid DC (pDC; *p* = 0.005), T helper cells (*p* = 0.003), and Th2 cells (*p* = 0.035), respectively.

To further evaluate the association between SLC2A1 and CRC samples immunofiltration cells, we analyzed the TIMER, GEPIA database, and TCGA CRC cohort to investigate the relationship between SLC2A1 and immunofiltration cells marker genes in various immunofiltration cells. As shown in Table 2, all investigations showed that SLC2A1 expression was correlated with neutrophil immune marker genes, including FCGR3A (r = 0.12, *p* = 4.6E-2), CD55 (r = 0.26, *p* = 1.1E-5) and ITGAM (r = 0.13, *p* = 2.6E-2). The scatter plots showed the correlation between SLC2A1 expression level and neutrophil immune marker genes, respectively (Figure 6D).

**TABLE 2:** Correlation analysis between SLC2A1 and immune cell marker gene in TIMER, GEPIA, and TCGA database.

Description	Gene markers	TIMER		GEPIA		TCGA	
		Purity		Tumor		Tumor	
		rho	*p*	rho	*p*	rho	*p*
B cell	CD19	0.063	2.06E-01	0.046	4.40E-01	–0.015	7.08E-01
	MS4A1	0.011	8.21E-01	–0.001	9.90E-01	–0.076	5.40E-02
	CD79A	0.049	3.21E-01	0.014	8.20E-01	0.003	9.30E-01
CD8 + T Cell	CD8A	0.030	5.43E-01	0.076	2.10E-01	0.003	9.31E-01
	CD8B	0.037	4.54E-01	0.051	4.00E-01	0.054	1.66E-01
	IL2RA	0.065	1.92E-01	0.110	8.00E-02	0.031	4.28E-01
Tfh	CXCR3	0.117	**1.81E-02**	0.130	**2.90E-02**	0.155	**7.62E-05**
	CXCR5	0.053	2.88E-01	–0.048	4.30E-01	0.033	3.98E-01
	ICOS	0.011	8.24E-01	0.079	1.90E-01	–0.042	2.89E-01
Th1	IL12RB1	–0.020	6.94E-01	0.015	8.10E-01	0.001	9.76E-01
	CCR1	0.126	**1.08E-02**	0.140	**2.20E-02**	0.076	5.34E-02
	CCR5	–0.017	7.25E-01	0.038	5.30E-01	–0.055	1.59E-01
Th2	CCR4	0.050	3.14E-01	0.049	4.20E-01	–0.009	8.28E-01
	CCR8	0.070	1.58E-01	0.090	1.40E-01	0.031	4.36E-01
	HAVCR1	0.060	2.29E-01	0.027	6.60E-01	0.074	6.13E-02
Th17	IL21R	0.108	**2.91E-02**	0.110	6.70E-02	0.019	6.35E-01
	IL23R	–0.044	3.79E-01	–0.085	1.60E-01	–0.152	**1.06E-04**
	CCR6	0.053	2.88E-01	0.095	1.20E-01	0.008	8.39E-01
Treg	FOXP3	0.059	2.36E-01	0.032	6.00E-01	0.043	2.71E-01
	NT5E	–0.009	8.54E-01	0.001	9.90E-01	–0.055	1.63E-01
	IL7R	0.082	1.00E-01	0.130	**3.70E-02**	0.000	9.96E-01
T cell exhaustion	PDCD1	0.085	8.57E-02	0.120	5.40E-02	0.111	**4.58E-03**
	CTLA4	–0.008	8.66E-01	0.053	3.80E-01	–0.027	4.88E-01
	LAG3	0.058	2.45E-01	0.082	1.70E-01	0.047	2.37E-01
M1 Macrophage	NOS2	0.072	1.47E-01	0.150	**1.50E-02**	0.068	8.61E-02
	IRF5	0.067	1.78E-01	0.098	1.10E-01	0.085	**2.98E-02**
	PTGS2	0.110	**2.73E-02**	0.170	**5.90E-03**	0.073	6.41E-02
M2 Macrophage	CD163	0.099	**4.58E-02**	0.110	6.80E-02	0.079	**4.50E-02**
	MRC1	0.122	**1.35E-02**	0.150	**1.30E-02**	0.065	1.01E-01
	CD209	0.060	2.29E-01	0.100	9.90E-02	0.042	2.84E-01
TAM	CCL2	0.024	6.25E-01	0.073	2.30E-01	0.034	3.92E-01
	CD86	0.033	5.03E-01	0.110	5.70E-02	0.023	5.67E-01
	CD68	0.208	**2.34E-05**	0.230	**1.30E-04**	–0.043	2.71E-01
Monocyte	CD14	0.131	**8.12E-03**	0.140	**2.00E-02**	0.152	**1.11E-04**
	CD33	0.040	4.20E-01	0.074	2.20E-01	0.056	1.55E-01
	ITGAX	0.164	**9.22E-04**	0.190	**1.30E-03**	0.094	**1.71E-02**
Natural killer cell	B3GAT1	0.085	8.86E-02	0.060	3.20E-01	0.031	4.34E-01
	KIR3DL1	0.091	6.79E-02	0.068	2.60E-01	–0.005	9.03E-01
	CD7	0.082	9.76E-02	0.120	**4.40E-02**	0.100	**1.07E-02**
Neutrophil	FCGR3A	0.069	1.68E-01	0.120	**4.60E-02**	0.047	2.37E-01
	CD55	0.167	**7.14E-04**	0.260	**1.10E-05**	0.118	**2.57E-03**
	ITGAM	0.119	**1.67E-02**	0.130	**2.60E-02**	0.096	**1.47E-02**
Dendritic cell	CD1C	–0.012	8.03E-01	–0.017	7.80E-01	0.000	9.97E-01
	THBD	0.134	**7.01E-03**	0.110	7.30E-02	0.125	**1.41E-03**
	NRP1	0.088	7.79E-02	0.150	**1.30E-02**	0.025	5.25E-01

Bold values indicate *p* < 0.05.

### Associations of SLC2A1 Expression Level With m6A Modification in CRC

**FIGURE 7:**
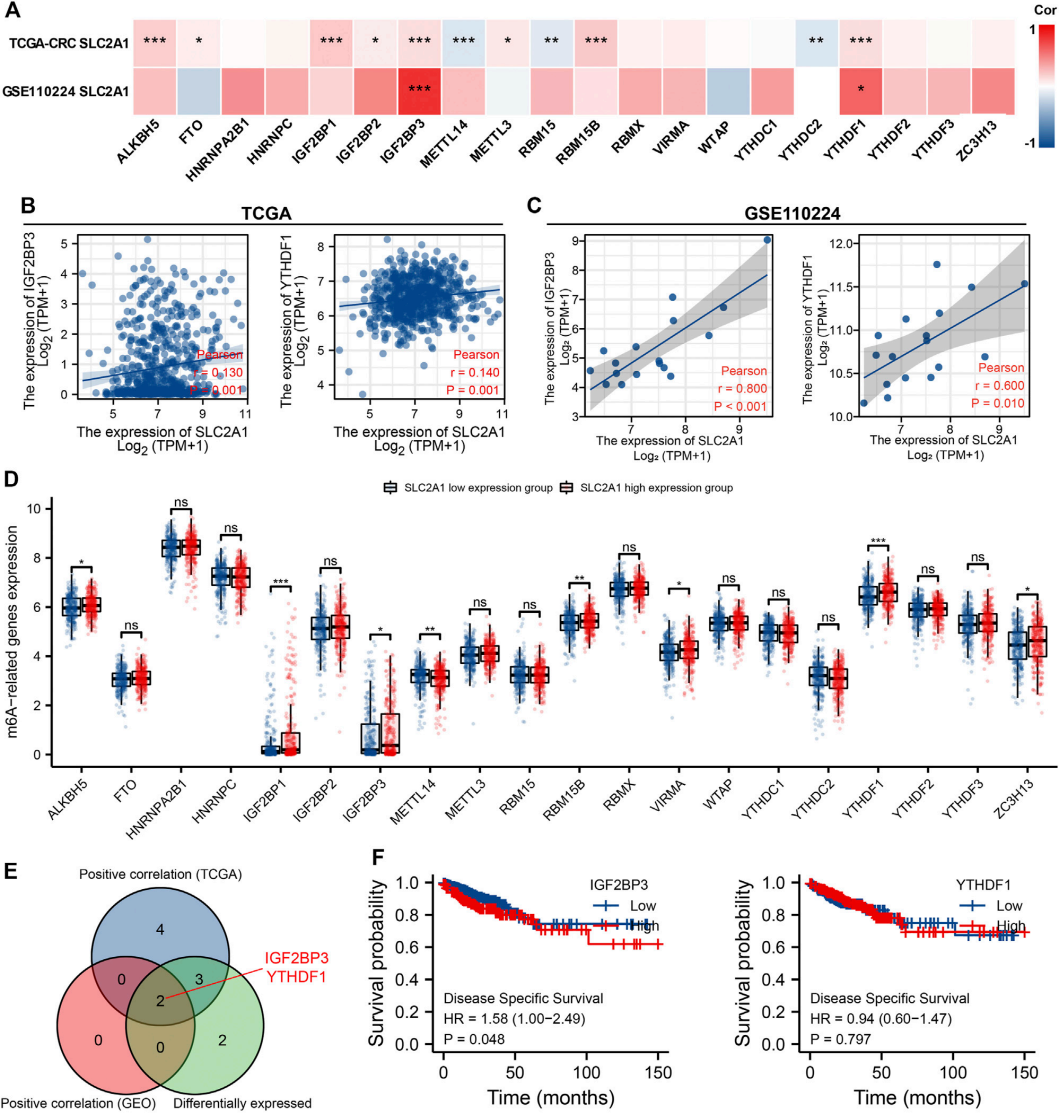
Associations of SLC2A1 expression with m6A relative genes in colorectal cancer (CRC). **(A)** GSE110224 and TCGA CRC cohort analyzed the association between the SLC2A1 and the 20 m6A relative genes expression in CRC. **(B)** TCGA CRC cohort was analyzed and a scatter plot was drawn to display the association between SLC2A1 and the expression of m6A relative genes, including IGF2BP3 and YTHDF1. **(C)** GSE110224 cohort was analyzed, and a scatter plot was drawn to display the association between SLC2A1 and the expression of m6A relative genes, including IGF2BP3 and YTHDF1. **(D)** The differential expression of 20 m6A relative genes between high and low SLC2A1 expression groups in CRC tumor samples. **(E)** Venn diagram indicated both expression association and differential expression of genes, including IGF2BP3 and YTHDF1. **(F)** The Kaplan–Meier curve of IGF2BP3 and YTHDF1. *, *p* < 0.05; **, *p* < 0.01; ***, *p* < 0.001; ****, *p* < 0.0001; ns, no significant.

The modification of m6A has a remarkable effect in the make progress of CRC. We analyzed GSE110224 and TCGA CRC cohort to explore the association between expression level of SLC2A1 and 20 m6A relative genes in CRC. As shown in Figure 7A, analysis indicated that, in the GSE110224 and TCGA CRC cohort, the SLC2A1 expression was remarkably positively associated with IGF2BP3 and YTHDF1 (*p* < 0.05). Furthermore, in the TCGA CRC cohort, SLC2A1 expression level was remarkably positively associated with ALKBH5, FTO, IGF2BP1, IGF2BP2, METTL3, and RBM15B (*p* < 0.05) but negatively associated with METTL14, RBM15, and YTHDC2 (*p* < 0.05).

As shown in Figures 7B,C, the scatter plot indicated the relationship between SLC2A1 and m6A relative genes. Furthermore, TCGA CRC samples were divided into high expression group and low expression group according to the SLC2A1 expression level. As shown in Figure 7D, we attempt to analyze the differential expression level of m6A relative gene between the high SLC2A1 expression group and low SLC2A1 expression group to confirm whether there is a difference in m6A modification between the two groups in CRC. The analyses indicated that, compared with the low expression group, the expression of ALKBH5, IGF2BP1, IGF2BP3, RBM15B, VIRMA, YTHDF1, and ZC3H13 increased in the SLC2A1 high expression group, whereas the expression of METTL4 decreased (*p* < 0.05). As shown in Figure 7E, the analysis indicated the key genes (IGF2BP3 and YTHDF1) that have expression correlation and differential expression relationship with SLC2A1. The Kaplan–Meier curves indicated that high IGF2BP3 expression was remarkably related with poor prognosis of CRC (*p* < 0.05, Figure 7F), whereas YTHDF1 was not related with poor prognosis of patients with CRC. These analyses point out that SLC2A1 may be strongly associated to the m6A modification of CRC, particularly through the regulating of IGF2BP3, and eventually impact the development and prognosis of CRC.

### Associations of SLC2A1 Expression Level With Ferroptosis in CRC

**FIGURE 8:**
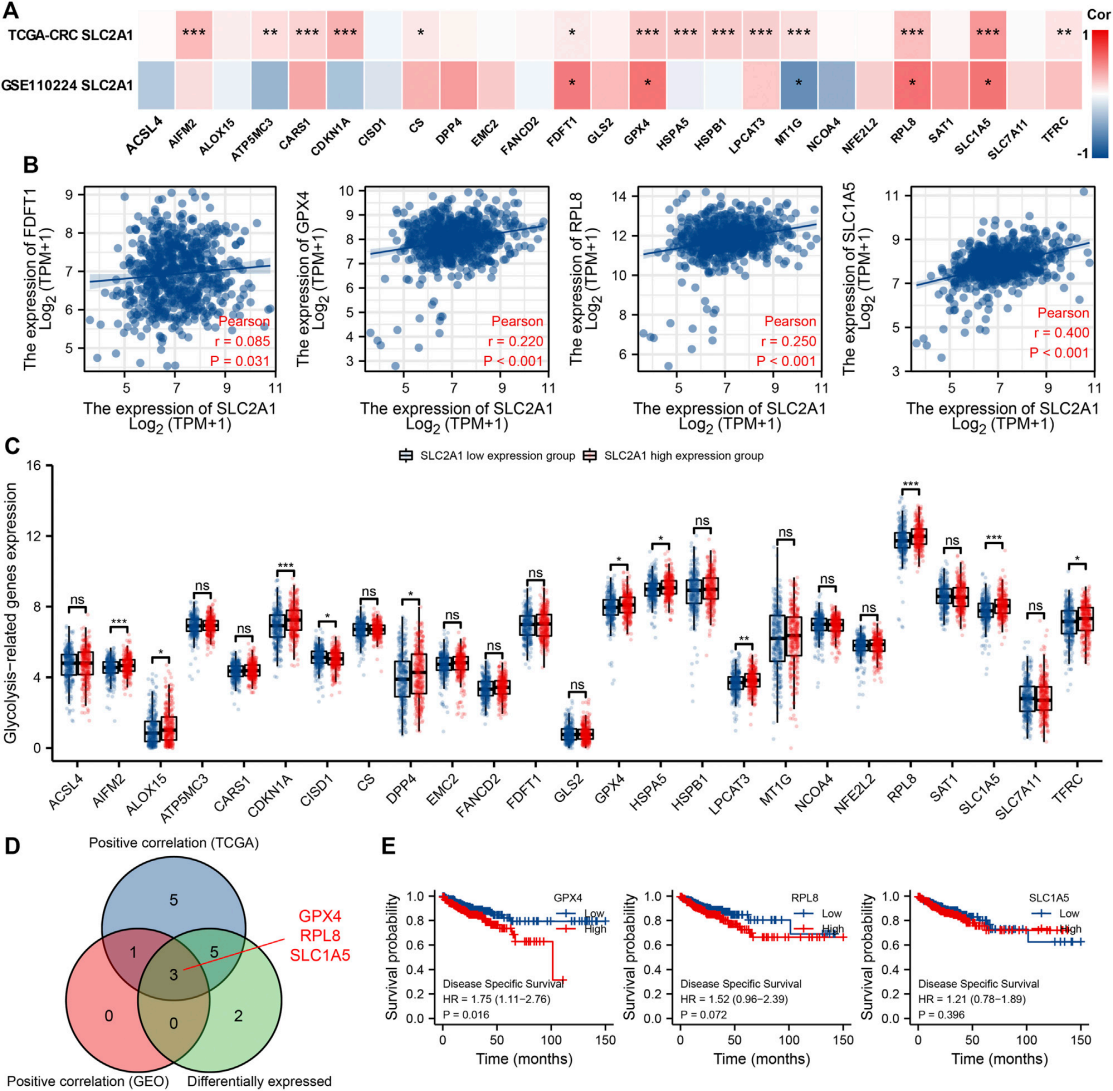
Associations of SLC2A1 expression with ferroptosis relative genes in colorectal cancer (CRC). **(A)** GSE110224 and TCGA CRC cohort analyzed the association between the SLC2A1 and the 25 ferroptosis relative genes expression in CRC. **(B)** TCGA CRC cohort was analyzed and a scatter plot was drawn to display the correlation between SLC2A1 and the expression of ferroptosis relative genes, including FDFT1, GPX4, RPL8, and SLC1A5. **(C)** The differential expression of 25 ferroptosis relative genes between high and low SLC2A1 expression groups in CRC tumor samples. **(D)** Venn diagram indicated both expression association and differential expression of genes, including GPX4, RPL8, and SLC1A5. **(E)** Kaplan–Meier curve of GPX4, RPL8, and SLC1A5. *, *p* < 0.05; **, *p* < 0.01; ***, *p* < 0.001; ****, *p* < 0.0001; ns, no significant.

We analyzed GSE110224 and TCGA CRC cohort to study the association between SLC2A1 and 25 ferroptosis relative genes in CRC in terms of expression level. The correlation results showed that in GSE110224 and TCGA, the expression of SLC2A1 was significantly correlated with FDFT1, GPX4, RPL8, and SLC1A5 (*p* < 0.05, Figure 8A). In addition, in the TCGA CRC cohort, SLC2A1 expression was positively correlated with AIFM2, ATP5MC3, CARS1, CDKN1A, CS, HSPA5, HSPB1, LPCAT3, MT1G, and TFRC (*p* < 0.05). In GSE110224 cohort, SLC2A1 expression was greatly negatively correlated with MT1G (*p* < 0.05).

The scatter graph demonstrated the correlation between SLC2A1 and ferroptosis of relevant genes (Figure 8B). In addition, according to the expression level of SLC2A1, TCGA CRC samples were set to high expression group and low expression group according to the SLC2A1 expression level. To analyze the differential expression levels of ferroptosis relative genes between the two groups in CRC (Figure 8C). The analyses indicated that, compared with the low expression group, the expressions of AIFM2, ALOX15, CDKN1A, DPP4, GPX4, HSPA5, LPCAT3, RPL8, SLC1A5, and TFRC increased in SLC2A1 high expression group, whereas the expression of CISD1 decreased (*p* < 0.05). As shown in Figure 8D, the analysis indicated the key genes (GPX4, RPL8, and SLC1A5) that have expression correlation and differential expression relationship with SLC2A1. The Kaplan–Meier curves indicated that high GPX4 expression was significantly correlated with poor prognosis in CRC (*p* < 0.05), whereas RPL8 and SLC1A5 expression were not (Figure 8E). These results point out that SLC2A1 may be significantly correlated to ferroptosis of CRC, especially through the regulation of GPX4, and eventually affect the development of CRC.

### Prediction and Construction of SLC2A1 ceRNA Regulatory Network in CRC

**FIGURE 9:**
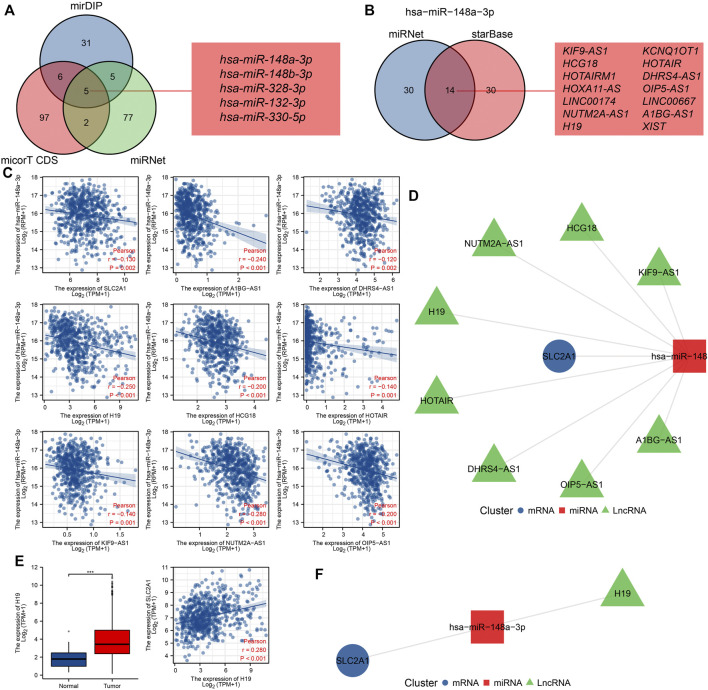
Prediction and construction of SLC2A1 ceRNA network in colorectal cancer (CRC). **(A)** The Venn database displays miRNA of targeted SLC2A1 predicted by the mirDIP, micorT CDS, and miRNet tools. **(B)** The Venn database displays lncRNA of targeted hsa-miR-148a-3p predicted by the miRNet and starBase tools. **(C)** Scatter plots show results with expressional correlations, including SLC2A1 and hsa-miR-148a-3p, hsa-miR-148a-3p and AKIF9-AS1, hsa-miR-148a-3p and DHRS4-AS1, hsa-miR-148a-3p and H19, hsa-miR-148a-3p and HCG18, hsa-miR-148a-3p and HOTAIR, hsa-miR-148a-3p and KIF9-AS1, hsa-miR-148a-3p and NUTM2A-AS1, and hsa-miR-148a-3p and OIP5-AS1. **(D)** The network diagram shows the relationship of the predicted ceRNA network. **(E)** Only H19 was highly expressed in CRC and positively associated with SLC2A1 expression. **(F)** The network diagram shows the relationship of the final ceRNA network.

Studies have shown that the regulatory mechanism of ceRNA plays a key role in CRC, so we attempted to predict and screen the ceRNA regulatory network of SLC2A1 in CRC. We predicted 47, 110, and 89 miRNAs targeting SLC2A1 using mirDIP, micorT CDS, and miRNet tools, respectively. Venn diagrams show the results predicted by the mirDIP, micorT CDS, and miRNet tools. As shown in Figures 5, 9A, miRNAs were predicted by the three tools, including hsa-miR-148a-3p, hsa-miR-148b-3p, hsa-miR-328-3p, hsa-miR-132-3p, and hsa-miR-330-5p. Furthermore, we analyzed the association between these miRNAs and SLC2A1 expression levels and found that only hsa-miR-148a-3p (r = −0.130, *p* = 0.002) was negatively correlated with SLC2A1 expression levels in CRC (Figure 9C).

We further predicted that the lncRNAs might bind to hsa-miR-148a-3p using miRNet and starBase tools, and the predicted results were the same as previous studies (Liu et al., 2021a). Venn diagrams show the results predicted by the miRNet and starBase tools. A total of 14 lncRNAs were predicted by both tools, including KIF9-AS1, HCG18, HOTAIRM1, HOXA11-AS, LINC00174, NUTM2A-AS1, H19, KCNQ1OT1, HOTAIR, DHRS4-AS1, OIP5-AS1, LINC00667, A1BG-AS1, and XIST (Figure 9B). Further analysis of the correlation between these lncRNAs and hsa-miR-148a-3p expression levels revealed that AKIF9-AS1 (r = −0.240, *p* < 0.001), DHRS4-AS1 (r = −0.120, *p* = 0.002), H19 (r = −0.250, *p* < 0.001), HCG18 (r = −0.200, *p* < 0.001), HOTAIR (r = −0.200, *p* < 0.001), KIF9-AS1 (r = −0.140, *p* < 0.001), NUTM2A-AS1 (r = −0.280, *p* < 0.001), and OIP5-AS1 (r = −0.200, *p* < 0.001) were negatively correlated with hsa-miR-148a-3p expression levels in CRC (Figure 9C). The network diagram shows the relationship of the predicted ceRNA network (Figure 9D).

To further narrow the prediction range, we analyzed the expression of target lncRNA and its correlation with SLC2A1 expression in the CRC cohort. The results showed that only H19 was upregulated in CRC (*p* < 0.001), and there was a significant positive correlation with SLC2A1 expression (r = 0.280, *p* < 0.001, Figure 9E). Therefore, we predict that the H19-hsa-miR-148a-3p-SLC2A1 ceRNA network may play a significant role in the development of CRC. The network diagram shows the relationship of the final ceRNA network (Figure 9F).

## Discussion

Glucose is one of the basic metabolites needed by animal and plant cells and the main energy source for tumor cell proliferation (Wang et al., 2020). Previous studies have confirmed that the GLUT1 protein encoded by SLC2A1 gene is mainly localized in the cell membrane. The expression level of GLUT1 in tumor cells is significantly higher than that in normal tissue cells, thus promoting the proliferation of tumor cells by enhancing the ability of glycolysis of tumor cells (Kasahara and Hinkle, 1977; Ancey et al., 2018). Studies have shown that the overexpression of SLC2A1 in cervical cancer (Rudlowski et al., 2004), gastric cancer (Berlth et al., 2015), esophageal cancer (Kato et al., 2002), and oral cancer (Pereira et al., 2013) can advance the proliferation of cancer cells. These analyses point out that SLC2A1 may be a possible target for cancer targeted therapy. Nevertheless, there are few research studies on the synthesis study of SLC2A1 in CRC.

In this project, we predicted the high expression of SLC2A1 in a variety of cancers by analyzing Oncomine database and TCGA cohort. Analysis showed that SLC2A1 was highly expressed in nine types of tumors in the Oncomine database, and analysis of the TCGA cohort showed that SLC2A1 was highly expressed in 15 types of tumors, which was consistent with the results of previous studies (Kato et al., 2002; Rudlowski et al., 2004; van Laarhoven et al., 2006; Pereira et al., 2013; Berlth et al., 2015; Avanzato et al., 2018). Through analysis of GEO and TCGA CRC cohort, it was found that the expression level of SLC2A1 in CRC samples was significantly higher than that in normal samples. The mRNA and protein levels of SLC2A1 in CRC samples were remarkably higher than those in the normal control group through *in vitro* experiment and IHC staining, and the analysis results are the same as the above studies. We also analyzed the ability of SLC2A1 expression to predict CRC by drawing ROC curves and found that SLC2A1 has high accuracy in predicting the outcomes of normal and tumor. At the same time, the SLC2A1 expression level was found to be associated to CRC tumors stage and progression free interval (PFI). Finally, SLC2A1 may serve as a possible diagnostic and therapeutic marker for CRC.

Presently, research studies on the role of SLC2A1 in tumors primarily focus on glucose transport and glycolysis, although there are few studies on other biological functions that may be involved in SLC2A1. In this study, the R statistical computing language was utilized to analyze the SLC2A1 co-expression genes in CRC, and it was discovered that the expression of EPHA2, KRT80, and KRT19 in CRC had the remarkable association with SLC2A1. Martini et al. (2019) suggested that EPHA2 was highly expressed in CRC and its expression level was closely related to PFI, suggesting that EPHA2 could be a potential therapeutic target for metastatic CRC. Li et al. (2018) pointed out that KRT80 is an independent prognostic biomarker for CRC and promotes CRC migration and invasion by activating the AKT pathway and interacting with PRKDC. Alsharif et al. (2021) suggested that KRT19 was highly expressed in breast cancer and could stabilize the E-cadherin complex on the cell membrane, maintain cell–cell adhesion, and provide growth and survival advantages for tumor cells. The GO term and KEGG pathway analysis of top 200 co-expressed genes positively associated with SLC2A1 expression indicated that these genes were mainly correlated to epidermis development, cell–cell junction, and cadherin binding. The KEGG enrichment analysis indicated that these genes were mainly related to Shigellosis and Wnt signaling pathway. GSEA indicated that the differential genes grouped according to SLC2A1 expression were primarily participated in REACTOME G ALPHA I SIGNALING EVENTS, REACTOME M PHASE, REACTOME NEURONAL SYSTEM, WP LNCRNA INVOLVEMENT IN CANONICAL WNT SIGNALING AND COLORECTAL CANCER, PID HIF1 TFPATHWAY, and REACTOME GLYCOLYSIS pathways. According to previous research studies, we found that the progression and metastasis of CRC are significantly correlated to the latter three pathways (Peng et al., 2020; Shen et al., 2021).

By the analysis of TIMER database, it was discovered that the expression level of SLC2A1 in CRC was negatively associated with the expression levels of B cell but positively associated with the expression levels of CD4+ T cell, neutrophil, and DC. Moreover, SLC2A1 CNV was remarkably associated with B cell, CD8+ T cell, neutrophil, and DC. These analyses point out that SLC2A1 maybe participated in the immune respond to CRC tumor microenvironment, particularly to B cell, neutrophil, and DC. The ratio of 24 tumor immune cells in CRC was detected by ssGSEA algorithm. We certified nine types of immune cells: DC, eosinophils, iDC, neutrophils, NK CD56 bright cells, NK cells, pDC, T helper cells, and Th2 cells, whose expression ratios showed considerable difference between the high and low SLC2A1 expression groups. Furthermore, through the analysis of three databases, we suggested that the expression level of SLC2A1 was remarkably positively associated with the gene markers of neutrophil, suggesting that SLC2A1 may influence the immunofiltration of SLC2A1 by affecting the expression of neutrophil. Neutrophil is an important immune cell in human body. Ning et al. (2015) found that excessive neutrophils can increase the expression of interleukin (IL)-1β and then induce the IL-17 response of CRC cells and support the occurrence of CRC. Wang et al. (2014) suggested that depletion of neutrophils or blocking of IL-1β activity significantly reduced mucosal damage and the formation of CRC tumors. We speculated that the overexpression of SLC2A1 promoted the infiltration of neutrophil in CRC and ultimately played an important role in promoting tumor proliferation. We pointed out that the high expression of SLC2A1 may enhance an anti-tumor immune response, stating that SLC2A1 plays a critical role in the immune regulation of CRC. Nevertheless, more prospective studies are needed to certify our speculation more accurately, including the relationship between SLC2A1 and neutrophil.

The modification of m6A is the prevalent common RNA modifications, which can affect tumor progression and metastasis by affecting the expression of several cancer-relative genes (Shen et al., 2020; Chen et al., 2021). On the project, we discovered that the SLC2A1 was remarkably positively associated with IGF2BP3 and YTHDF1. We also discovered that, in the group with high SLC2A1 expression, the expression levels of ALKBH5, IGF2BP1, IGF2BP3, RBM15B, VIRMA, YTHDF1, and ZC3H13 significantly increased. Yang et al. (2020) found that downregulation of IGF2BP3 could affect the expression of CCND1 and VEGF and thus inhibit DNA replication and angiogenesis in the S phase of tumor cell cycle. Bai et al. (2019) found that YTHDF1 was overexpressed in CRC, and knockdown of YTHDF1 expression could significantly inhibit the tumorigenicity of CRC cells *in vitro* and the growth of xenograft tumors in mice *in vivo*. Therefore, we speculate that SLC2A1 may interact with IGF2BP3 and YTHDF1 in CRC and ultimately affect the occurrence and development of CRC. Eventually, the Kaplan–Meier survival analysis of patients with CRC indicated that high IGF2BP3 expression correlated with significantly worse patient survival. We suggest that the promoting effect of SLC2A1 on CRC may be associated to m6A modification, and SLC2A1 can impact the CRC methylation level through its correlation with IGF2BP3 and finally impact the development and prognosis of CRC.

To grow, cancer cells show a higher need for iron than normal cells. This dependence on iron can make cancer cells more susceptible to iron-mediated necrosis, known as iron death (Ma et al., 2018). In this project, we discovered that the expression level of SLC2A1 was remarkably positively associated with ferroptosis relative genes, including FDFT1, GPX4, RPL8, and SLC1A5. We also found that, in the group with high SLC2A1 expression, the expression levels of AIFM2, ALOX15, CDKN1A, DPP4, GPX4, HSPA5, LPCAT3, RPL8, SLC1A5, and TFRC significantly increased. Eventually, the Kaplan–Meier survival analysis of patients with CRC indicated that high GPX4 expression correlated with significantly worse patient survival. Yang et al. (2021) found that cell ferroptosis may be inhibited in CRC cells through KIF20A/NUAK1/PP1β/GPX4 pathway, which may be the basis of oxaliplatin resistance. Therefore, we speculate that, in CRC, SLC2A1 may interact with GPX4 and finally affect the development of CRC. We suggest that the promoting effect of SLC2A1 on CRC may be associated to the mechanism of ferroptosis of tumor cells. SLC2A1 can achieve the inhibition of CRC ferroptosis by promoting the expression of GPX4 and ultimately promote the development of CRC.

The ceRNA network plays an important role in the development of cancer, and the theory of its role is mainly realized through the competitive binding of lncRNA or circular RNA to miRNA, which ultimately affects mRNA expression level (Salmena et al., 2011). Zhao et al. (2021) found that lncRNA MIR17HG acted as a ceRNA to regulate the expression of HK1 through sponging miR-138-5p, leading to glycolysis of CRC cells, leading to its invasion and liver metastasis. In this project, we first predicted five potential upstream miRNAs jointly through three databases, but only one miRNA (hsa-miR-148a-3p) was negatively relationship with SLC2A1 expression in CRC. Nersisyan et al. (2021) found that the downregulation of hsa-miR-148a-3p led to the upregulation of its two target genes, ITGA5 and PRNP, ultimately promoting the progression of CRC and leading to low survival rate in patients with CRC. Then, we further predicted the upstream lncRNAs of hsa-miR-148a-3p, and obtained 14 potential upstream lncRNAs. Correlation analysis showed that only eight lncRNAs expressed in CRC were negatively correlated with hsa-miR-148a-3p expression. To further narrow the scope of the ceRNA network, we analyzed the expression of eight lncRNAs in CRC and the correlation between their expression and SLC2A1 and found that only lncRNA H19 was highly expressed in CRC and positively correlated with SLC2A1 expression level. Zhang et al. (2020) found that lncRNA H19 promoted the transfer of junction CRC by binding with hnRNPA2B1. These studies further suggested the feasibility of our analysis. lncRNA H19 sponges mediated hsa-miR-148a-3p to regulate the expression of SLC2A1, thereby promoting the glycolysis and proliferation of CRC. However, although the ceRNA network of SLC2A1 has been obtained through a large number of database analyses, more basic studies are needed to further prove our analysis.

In conclusion, our study is the first one to analyze the association between SLC2A1 expression and immune invasion, m6A modification, ferroptosis, and ceRNA regulatory network in CRC from multiple perspectives. m6A can enhance the stability of SLC2A1 mRNA by modifying SLC2A1 gene, thus promoting the glycolysis and cell proliferation of CRC. SLC2A1 expression is associated with various immune infiltration cells and may influence CRC tumor immune microenvironment by promoting neutrophil infiltration. In the correlation analysis of ferroptosis, it was found that SLC2A1 might promote the expression of GPX4 and then inhibit the ferroptosis of CRC. The construction of SLC2A1 ceRNA network suggests that lncRNA H19/hsa-miR-148a-3p/SLC2A1 ceRNA network may promote the development of CRC. SLC2A1 could be considered as a potential biomarker for the diagnosis and treatment of patients with CRC. However, most of the results in this paper are obtained by bioinformatic analysis. A series of experiments will be carried out to further validate these results in the future, clarifying that the high expression of SLC2A1 can have the role of diagnostic CRC and that interference with SLC2A1 expression can achieve the purpose of treating tumors.

## Data Availability

The datasets presented in this study can be found in online repositories. The names of the repository/repositories and accession number(s) can be found in the article/Supplementary Material.
