# Supplementary material for: SLC2A1 is a Diagnostic Biomarker Involved in Immune Infiltration of Colorectal Cancer and Associated With m6A Modification and ceRNA
Source: Front Cell Dev Biol. 2022 Mar 24;10:853596. doi: 10.3389/fcell.2022.853596 (PMC8987357; doi:10.3389/fcell.2022.853596)
Supplement: Supplementary file 4 [file DataSheet1.DOCX]

Supplementary Material

# Supplementary Methods

## Cell Lines and Cell Culture Reagents

Human colorectal cancer (CRC) cell lines SW480 and HCT116 and normal human colorectal mucosa cells line FHC were obtained from the American Type Culture Collection (Manassas, VA, USA). The cells were maintained in DMEM high glucose medium (Hyclone, Logan, UT, USA) supplemented with 10% FBS (Gibco, USA) and 1% antibiotics (penicillin-streptomycin, Gibco, USA).

## RNA Extraction and qRT-PCR

The implementation method refers to previous study. Total RNA was isolated from cells using Trizol reagent (Invitrogen, Carlsbad, CA, USA). Use Prime Script RT reagent kit (Takara, Dalian, China) for reverse transcription, and then use SYBR Prime Script RT PCR kit (Takara, Dalian, China) for qRT-PCR. Use GAPDH as an internal reference and use the 2^-△△Ct^ method to calculate the results. SLC2A1 primer sequences: forward primer CTTTGTGGCCTTCTTTGAAGT and reverse primer CCACACAGTTGCTCCACAT. GAPDH primer sequences: forward primer GGAGCGAGATCCCTCCAAAAT and reverse primer GGCTGTTGTCATACTTCTCATGG.

## Immunohistochemistry

Clinical samples were obtained from 48 patients with CRC who were surgically treated at Taihe Hospital Affiliated of Hubei University of Medicine from July 2018 to December 2019. The content of SLC2A1 was detected by IHC according to the method previously described. The CRC tissue and the paracarcinoma tissues were prepared into 3 μm paraffin sections and incubated with mouse monoclonal antibodies of SLC2A1 (1:200, Abcam, USA) at 4℃ overnight in a refrigerator. The sections were coupled with the goat anti-mouse IgG-HRP secondary antibody (1:2000, Abcam, USA) at room temperature for 1.5 h, then each incubated section was stained with DAB reagent, and finally counterstained with hematoxylin.

IHC staining scores of SLC2A1 were assessed by two experienced observers. IHC score of tumor cells was 0-3: 0, negative; 1, weak; 2, medium; 3, strong.

# Supplementary Tables

## Supplementary Table 1. SLC2A1 co-expressed genes

## Supplementary Table 2. The GO and KEGG enrichment analysis of SLC2A1 co-expression genes

## Supplementary Table 3. SLC2A1 GSEA in CRC
